# Current Researches, Rationale, Plausibility, and Evidence Gaps on Metformin for the Management of Hypertensive Disorders of Pregnancy

**DOI:** 10.3389/fphar.2020.596145

**Published:** 2020-12-14

**Authors:** Yang Zhang, Xiaoxia Liu, Liu Yang, Li Zou

**Affiliations:** Department of Obstetrics and Gynecology, Union Hospital, Tongji Medical College, Huazhong University of Science and Technology, Wuhan, China

**Keywords:** metformin, hypertensive disorders of pregnancy (HDP), preeclampsia, prevention, treatment

## Abstract

Hypertensive disorders of pregnancy (HDP) are a group of morbid pregnancy complications, with preeclampsia (PE) being the most common subclassification among them. PE affects 2%–8% of pregnancies globally and threatens maternal and fetal health seriously. However, the only effective treatment of PE to date is the timely termination of pregnancy, albeit with increased perinatal risks. Hence, more emerging therapies for PE management are in urgent need. Originally introduced as the first-line therapy for type 2 diabetes mellitus, metformin (MET) has now been found in clinical trials to significantly reduce the incidence of gestational hypertension and PE in pregnant women with PE-related risks, including but not limited to pregestational diabetes mellitus, gestational diabetes mellitus, polycystic ovary syndrome, or obesity. Additionally, existing clinical data have preliminarily ensured the safety of taking MET during human pregnancies. Relevant lab studies have indicated that the underlying mechanism includes angiogenesis promotion, endothelial protection, anti-inflammatory effects, and particularly protective effects on trophoblast cells against the risk factors, which are beneficial to placental development. Together with its global availability, easy administration, and low cost, MET is expected to be a promising option for the prevention and treatment of PE. Nevertheless, there are still some limitations in current studies, and the design of the relevant research scheme is supposed to be further improved in the future. Herein, we summarize the relevant clinical and experimental researches to discuss the rationale, safety, and feasibility of MET for the management of HDP. At the end of the article, gaps in current researches are proposed. Concretely, experimental MET concentration and PE models should be chosen cautiously. Besides, the clinical trial protocol should be further optimized to evaluate the reduction in the prevalence of PE as a primary endpoint. All of those evidence gaps may be of guiding significance to improve the design of relevant experiments and clinical trials in the future.

## Introduction

Hypertensive disorders of pregnancy (HDP), a group of morbid pregnancy complications, include gestational hypertension, preeclampsia (PE), eclampsia, and chronic hypertension in pregnancy (Cushen and Goulopoulou, 2017). Among them, PE is the most common subclassification characterized by new-onset hypertension typically after 20 weeks of gestation, accompanied by multisystem signs or symptoms, including proteinuria, elevated liver enzymes, renal insufficiency, thrombocytopenia, pulmonary edema, persistent severe headache, seizures (eclampsia), and even maternal and fetal death (Duley, 2009). PE is one of the leading causes of maternal and perinatal morbidity and mortality (ACOG, 2019a; ACOG 2019b). Reportedly, PE complicates 2%–8% of pregnancies globally (Steegers et al., 2010), accounting for 16% of maternal mortality worldwide (Khan et al., 2006; Ronsmans and Graham, 2006) with most of them occurring in low- and middle-income countries, and is the most frequently cited indication for iatrogenic preterm labor in developed countries (Duley, 2009). It is now established that PE not only elevates the risk of cardiovascular and metabolic diseases in mothers in subsequent years (Grandi et al., 2017; Wu et al., 2017; Abbasi, 2018) but also has long-term effects on cardiometabolic health (Davis et al., 2012; Lawlor et al., 2012; Davis et al., 2015; Rice et al., 2018) and neurodevelopment (Sun et al., 2020) of the offspring, leaving the prevention and cure of PE a matter of great urgency. Regrettably, the exact etiopathogenesis of PE remains vague and complicated, leading to a clinical dilemma where low-dose aspirin is merely used for prevention and antihypertensive and antispasmodic drugs are mainly symptomatic treatments, while thus far, the only curative intervention for PE is the delivery of the fetus and placenta, albeit with raised consequent risks (Rolnik et al., 2017). Hence, more possible therapeutic strategies remain to be further explored.

Emerging progress in understanding the preeclamptic pathogenesis leads to an interest in more novel agents to prevent and treat PE, among which metformin (MET) has preliminarily proved to be safe during human pregnancies and effective in the prevention or treatment of PE. Well known as the first-line therapy for type 2 diabetes mellitus (Bailey, 2017), MET, in the area of reproduction and pregnancy, has been frequently used to improve ovulation in the treatment of polycystic ovary syndrome (PCOS) and recommended as an alternative to insulin in women with gestational diabetes mellitus (GDM) (Lazaridou et al., 2017; Lindsay and Loeken, 2017). Relevant clinical trials have reported that MET shows preventive effects on gestational hypertension and PE in pregnant women with high risks, including but not limited to pregestational diabetes mellitus (PGDM), GDM, PCOS, and obesity (Balsells et al., 2015; Jiang et al., 2015; Brownfoot et al., 2016; Syngelaki et al., 2016). In the meantime, an increasing number of lab studies have revealed that MET may have preventive and curative properties for PE via angiogenesis promotion, endothelial protection, anti-inflammatory effects, and protective effects on trophoblast cells in particular. Therefore, MET is expected to be a promising therapy in PE, though more research and more extensive trials are required. In this article, we will discuss the latest theories of preeclamptic etiopathogenesis, review the existing clinical research reporting the preventive or therapeutic properties of MET in HDP, and summarize the lab studies in this field to suggest the putative mechanisms. Furthermore, we will end the article by highlighting some gaps in the available evidence and some unsettled questions related to the prospect of the research direction in the future.

## Newest Theories of Preeclampsia Pathogenesis

**FIGURE 1:**
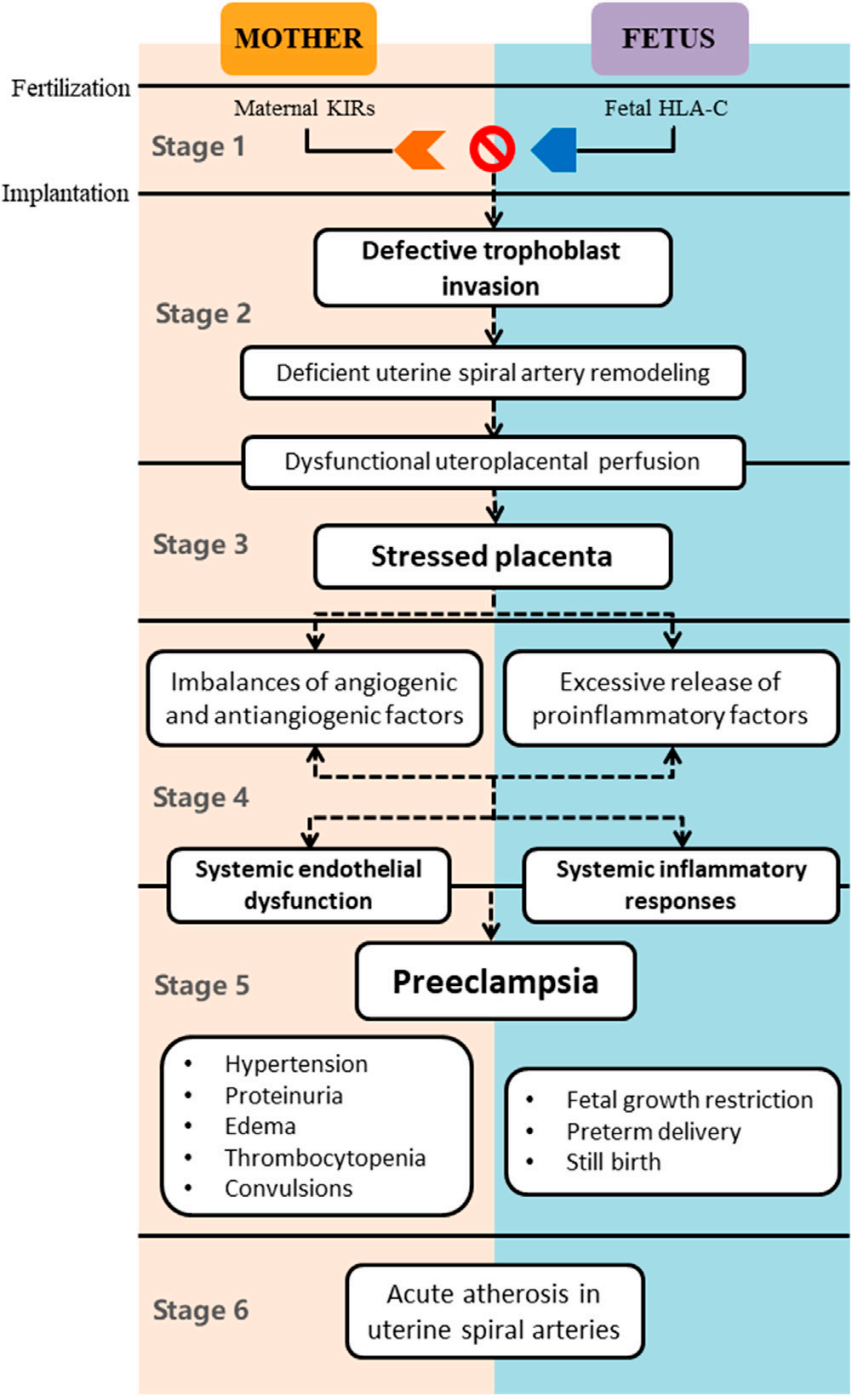
Six-stage theory of PE pathogenesis.

Previously, the classic two-stage theory of PE proposed by Redman has been widely accepted; that is, the first stage mainly comprises poor placentation, resulting in the excessive release of antiangiogenic and proinflammatory factors into the maternal circulation, which is the reason for multiple clinical manifestations of the disease in the second stage (Redman and Sargent, 2009). With a rapid increase in knowledge, the two-stage model has become inadequate; thus, Redman et al. newly came up with the six-stage theory to further expound the pathogenesis of PE (Figure 1) (Redman, 2014). The first stage is immunological in origin. The maternal immune tolerance to the allograft is essential for productive pregnancy since the fetus is regarded as a semiallograft. There is a short interval from fertilization to embryo implantation, during which uterine NK cells (uNK) are the main maternal leukocytes participating in the embryo implantation. uNK highly express the killer immunoglobulin-like receptors (KIRs), which bind to the HLA-C molecules on trophoblast cells. The inappropriate interactions between the KIRs encoded via maternal genes and the HLA-C encoded via fetal genes play determinant roles in the pathogenesis of PE and other placentally related complications. In other words, the mother failing to develop immune tolerance to the paternal genes of the embryo is more likely to have defective placentation (Saito et al., 2007). The second stage, 8–18 weeks of gestation, is the critical period of placentation mainly initiated with the trophoblast-mediated remodeling of uterine spiral arteries. Sufficient remodeling contributes to the dilation of the terminal segments of the uterine spiral arteries, thus decreasing the vascular resistance. The fall in the spiral artery resistance reduces the velocity and pulsatility of the inflowing maternal blood, which has protective effects on the delicate placental villi and microvilli. However, trophoblast dysfunction, especially the impaired invasive ability, is closely linked with PE, inducing the deficient remodeling of uterine spiral arteries. Abnormal remodeling leads to the dysfunctional perfusion of the intervillous space with hemodynamic stress and oxygenized arterial blood, thus suppressing the growth and development of the villous tree in the third stage, also called the placental stress stage. The fourth stage represents the imbalances of angiogenic and antiangiogenic factors derived from the stressed placenta. The excessive secretion of antiangiogenic substances, typically including soluble fms-like tyrosine kinase-1 (sFlt-1) and soluble endoglin (sENG), and the deficient production of angiogenic factors, such as vascular endothelial growth factor (VEGF) and placental growth factor (PlGF) (Levine et al., 2006; Venkatesha et al., 2006; Roberts, 2014; Zeisler et al., 2016; Herraiz et al., 2018), jointly induce the basic pathological changes of PE: maternal systemic endothelial dysfunction and arteriole spasm. Meanwhile, uteroplacental and systemic inflammatory responses also play a key role in this stage, characterized by the immoderate release of proinflammatory factors from the stressed placenta into the maternal blood, exacerbating the maternal multiple organ dysfunction and systemic inflammatory responses. Once the diagnosis of PE can be clinically made, the fifth stage has been confirmed. No more than 10% of patients with PE will experience the sixth stage (Kim et al., 2015), at which the remolded spiral arteries become rapidly atherosclerotic in the placental bed and the decidual nontransformed arteries. Acute atherosclerosis in spiral arteries further limits the uteroplacental perfusion, exacerbated by the spiral artery thrombosis, inducing the placental infarction with risks of fetal demise (Kim et al., 2015). Therefore, the underlying pathogenesis of PE has a variety of important features or elements, including but not limited to genetics, autoimmunity, inflammation, angiocardiopathy, defective placentation, and oxidative stress, any of which may not only be independent factors but also have intricate interactions with each other. Furthermore, it seems that the existing therapies of PE are not able to cover the complexity and diversity of PE (Tranquilli et al., 2013), and more novel therapies like MET are urgently needed.

## Progress of Clinical Research on Metformin in Preeclampsia

### Preventive and Therapeutic Effects of Metformin on Preeclampsia

Pregnancy complicated with diabetes comprises PGDM and GDM. PGDM is a risk factor for PE (Bartsch et al., 2016) and women suffering from GDM also have a risk of developing PE (ACOG, 2018). In a randomized, open-label study of pregnant women with PGDM, there were less gestational hypertension (*p* = 0.029) and maternal weight gain (*p* < 0.001) in the MET-treated group compared to the insulin-treated group (Ainuddin et al., 2015). In a case-control study carried out in pregnant women with GDM matched via weight, age, and ethnicity, maternal and neonatal outcomes were compared between the 100 women treated with insulin and the 100 women who remained exclusively on MET. Rates of gestational hypertension were similar in the two groups, whereas PE occurred less frequently in the MET group; however, there was no significant difference between the insulin and the MET groups. However, the incidence of PE was statistically less frequent once 13 patients requiring additional insulin were included with the MET group. Additionally, women treated with MET had significantly less mean weight gain from enrollment to term as high maternal weight is also a risk factor for PE (Balani et al., 2009). A meta-analysis of relevant randomized controlled trials (RCTs) comparing pregnancy outcomes in women with GDM treated with either MET or insulin showed that there was no significant difference in the PE rate between the two groups, whereas the incidence of gestational hypertension was significantly less in the MET group (OR = 0.52, *p* = 0.02). Likewise, average weight gains after enrollment were much lower in the MET group (Gui et al., 2013). Butalia et al. also found that MET was significantly superior to insulin in reducing the incidence of gestational hypertension and total maternal pregnancy weight gain in patients with GDM (Butalia et al., 2017). Presently, existing results show that MET is more likely to have preventive effects on gestational hypertension rather than PE; however, the rationale behind the development of gestational hypertension, as opposed to PE, is vague. Furthermore, both conditions may have overlapping processes or even have no essential difference (Okonkwo and DiPietro, 2017). Therefore, it is plausible to say that MET can also have an effect on PE in pregnancy complicated with diabetes, though more relevant research is warranted to confirm this inference.

Increased prepregnancy body mass index (BMI) is associated with increased risks of PE, particularly those women with prepregnancy BMI >30 kg/m^2^ (Burton et al., 2019). In a randomized clinical trial (Nascimento et al., 2020), obese pregnant women with a BMI greater than or equal to 30 kg/m^2^ were divided into two groups: the MET-treated group (*n* = 127) with a daily dose of 1.0 g and the control group (*n* = 145). All pregnant women entered the study with a gestational age of less than 20 weeks and were followed throughout the prenatal period. In the results, the incidence of PE was lower in the MET group compared to the control (6.3% vs. 21.4%, *p* < 0.01). Similarly, in a double-blind, placebo-controlled trial, the researchers randomly assigned nondiabetic women who had a BMI of more than 35 kg/m^2^ to receive MET or placebo (225 women in each group) from 12 to 18 weeks of gestation until delivery. MET was initiated at a daily dose of 1.0 g, and the dose was increased by 0.5 g per week to the maximum daily dose of 3.0 g. The incidence of PE was lower in the MET group (3.0% vs. 11.3%; RR = 0.24, *p* = 0.001), as was the median maternal gestational weight gain (Syngelaki et al., 2016). Another randomized, double-blind, placebo-controlled trial involved pregnant women with normal glucose tolerance who had a BMI of more than 30 kg/m^2^ and examined the effect of MET at a dose of 0.5 g daily (increasing to a maximum of 2.5 g) or matched placebo per day from between 12 and 16 weeks of gestation until delivery. Although the study showed no significant differences between the MET and placebo groups in the rate of PE, the inflammatory markers IL-6 and CRP were both significantly lower in women given MET (Chiswick et al., 2015). The MET-associated reduction in CRP and IL-6 might be beneficial since those inflammatory markers have been associated with adverse outcomes such as preterm birth and PE (Xu et al., 2014). Differences in trial design and compliance are the most likely explanations for the contradictory outcomes between the above two clinical trials. In the former trial, women had a higher BMI, received a higher starting and maximum dose of MET, and were more compliant. Conversely, poor compliance and underdose administration might be part of the reasons for the negative results in the latter.

Pregnancies in women with PCOS are characterized by more frequent complications, including gestational hypertension, PE, GDM, early pregnancy loss, preterm births, and neonatal admission to intensive care units. De Leo et al. (2011) conducted a prospective clinical trial to evaluate the safety and effect of MET during pregnancy in patients with PCOS. In this study, 98 pregnant women with PCOS and hyperinsulinemia were treated with MET (1.7–3.0 g/day) throughout pregnancy and 110 were normal pregnant controls. MET treatment in the pregnant patients with PCOS resulted in significantly lower rates of gestational hypertension (0% vs. 10.4%, *p* = 0.005), GDM, and miscarriage and a nonsignificant decrease in PE (0% vs. 2.08%, *p* = 0.24), compared to the control group. Similarly, another randomized, placebo-controlled, double-blind, multicentre clinical trial also showed that MET induced a nonsignificant decrease in the incidence of PE (3.0% vs. 7.0%; OR = 0.46; 95% CI, 0.17–1.15, *p* = 0.10) in pregnant women with PCOS, compared to the placebo-treated group (Løvvik et al., 2019).

Recently, Nascimento et al. (2018) conducted a meta-analysis to explore the influence of MET on PE. For the randomized studies of obese pregnant women, the MET treatments indicated a reduction of 49% in the risk of PE, and there was no significant decrease in gestational hypertension incidence. In the nonrandomized studies of pregnant women with PCOS, the result showed a possible 63% risk reduction for gestational hypertension with MET administration. For those randomized with PCOS, there was no risk reduction for PE and gestational hypertension. The randomized investigations of diabetic pregnant women indicated a 47% risk reduction for gestational hypertension compared with insulin; however, there was no significant reduction for PE.

### Safety

Currently, it is recommended that the dosage of MET for pregnant women usually starts at 0.5 g/day and then gradually increases to 2.5–3.0 g per day. The most common adverse effects of MET involve nausea, vomiting, diarrhea, and abdominal discomfort, which can be ameliorated by slowly increasing the dosage, taking the medication with meals, or switching to extended-release MET tablets. As MET is directly excreted in the prototype by the kidneys, it is prone to accumulate when renal function is impaired. Therefore, patients with renal insufficiency or hypoxemia should perform periodic inspection of the serum creatinine, creatinine clearance rate, blood urea nitrogen, and blood lactic acid before and during taking the MET to ensure decent renal function (ACOG, 2018). MET can freely cross the placental barrier, and fetus concentrations can achieve at least 50% of the maternal concentration. MET can be excreted into breast milk as well; however, the amount is clinically insignificant. Most trials comparing insulin with MET have not reported an increase in adverse perinatal outcomes associated with MET (Priya and Kalra, 2018). A five-year retrospective study showed that the initiation of MET within the first trimester of GDM women had no significant adverse maternal or fetal outcomes (Vanlalhruaii et al., 2018). In a meta-analysis in pregnant women with PCOS, MET showed the advantage of reducing the incidence of early pregnancy loss, preterm delivery, GDM, and gestational hypertension, without increasing the risk of teratogenicity or other serious side effects (Zeng et al., 2016). In the “MET in Gestational diabetes” (MiG) trial, pregnant women with GDM were randomly assigned to receive either insulin or MET (plus insulin if required). In the MET group, 46.3% of the women received supplemental insulin, and the perinatal complications did not differ between the two groups (Rowan et al., 2008). Some offspring of the subjects enrolled in the MiG trial were followed up to compare body composition and metabolic outcomes at 2 and 7–9 years of age. In the MET group, children had significantly larger mid-upper arm circumferences (17.2 ± 1.5 vs. 16.7 ± 1.5 cm; *p* = 0.002), biceps skinfolds (6.03 ± 1.9 vs. 5.6 ± 1.7 mm; *p* = 0.04), and subscapular skinfolds (6.3 ± 1.9 vs. 6.0 ± 1.7 mm; *p* = 0.02) at age 2, than those in the insulin group. However, the total fat mass and percentage of body fat assessed by bioimpedance showed no difference (Rowan et al., 2011). Furthermore, at 7–9 years of age, the follow-up study in the offspring of women in MiG showed that the total and abdominal body fat proportion and metabolic measures were similar between the two groups. However, at 9 years, offspring of MET-treated women were larger by measures of weight (37.0 ± 12.6 vs. 32.7 ± 7.7 kg; *p* = 0.049), waist (69.1 ± 12.2 vs. 64.2 ± 8.4 cm, *p* = 0.04) and arm (23.0 ± 4.3 vs. 21.2 ± 2.9 cm, *p* = 0.02) circumference, and the ratio of waist to height (0.51 ± 0.08 vs. 0.47 ± 0.05, *p* = 0.02) (Rowan et al., 2018). Those results indicated that the offspring exposed to MET in utero had larger BMI and subcutaneous fat than the nonexposed group; in other words, they had a higher risk of being overweight or obese. Nevertheless, it remains to be further explored whether this outcome is due to the effect of the MET or some women in the MET group may have reduced their energy intake, leading to relative “nutritional deficiencies” in the fetus, thus affecting the metabolic status of offspring. Relevant animal experiments have suggested that exposure to MET in early pregnancy may exert a negative impact on the reproductive system of male offspring (Salomäki et al., 2013). It is said that humans are more sensitive to MET than mice; however, there has been no evidence of negative impact in current studies yet (Carlsen and Vanky, 2010; Vanky and Carlsen, 2012; Tertti et al., 2016). Notably, the number of subjects in those studies was limited and the follow-up time was short. Besides, some studies only used physical examination to evaluate the development of the reproductive function. In the future, more cohort studies are required to explore the impact of intrauterine exposure to MET on the reproductive health of offspring from physical examination, hormone detection, and other aspects. Similarly, related researches have suggested that intrauterine MET exposure has no adverse effect on neuropsychological development in offspring (Glueck et al., 2004; Wouldes et al., 2016). However, given the limited number of current studies, more large-sample studies are still warranted in the future. Moreover, by adopting a high-reliability and high-validity scale for the offspring, the effects of intrauterine MET exposure on the neuropsychological development at different ages need to be accurately assessed.

Currently, most studies suggest that metformin has no negative effects on perinatal outcomes and newborns, and the American College of Obstetricians and Gynecologists (ACOG) indicated that MET could be reasonably used in pregnancy (ACOG, 2018). However, according to the “Developmental Origins of Health and Diseases” theory, the long-term effect of intrauterine MET exposure on the growth, metabolism, neuropsychological, and even reproductive function development of offspring still needs continuous attention; in other words, MET should be used with caution in pregnancy.

## Proposed Mechanisms by Which Metformin May Prevent and Cure Preeclampsia

### The Protective Effect of Trophoblasts Exerted by Metformin

According to the above-mentioned Redman’s theories, defective placentation plays a crucial role in the pathogenesis of PE, with trophoblast dysfunction regarded as the major trigger (Chaiworapongsa et al., 2014). Due to varieties of risk factors, increased apoptosis or necrosis (Leung et al., 2001; Crocker et al., 2003) and particularly restricted invasion of trophoblast cells (Strickland and Richards, 1992; Cross et al., 1994; Conrad and Benyo, 1997) lead to shallow implantation and compromised spiral artery remodeling, thus initiating the subsequent physiopathologic changes of PE. Therefore, a drug that can protect the function of trophoblast cells against high risks may be an attractive candidate for tackling the root of the problem.

Wang et al. (2014) found that upregulated expression of the receptor for the globular head of human C1q (gC1qR) in trophoblast cell lines HTR-8/SVneo cells induced apoptosis and mitochondrial dysfunction, while MET stabilized mitochondrial function and abrogated the increase of cellular apoptosis induced by the overexpression of gC1qR. Endoplasmic reticulum stress (ERS) in trophoblast has been implicated in the pathophysiology of PE. Immoderate levels of ERS reduce cell proliferation and stimulate the release of antiangiogenic factors and proinflammatory cytokines, increasing the risks of PE (Burton et al., 2017). In BeWo cells induced by ERS inducer, MET showed protective effects via suppressing ERS in BeWo cells. Besides, MET restored PlGF levels reduced by high levels of cellular ERS, which might also be beneficial to the prevention and treatment of PE as PlGF is reduced particularly in early-onset PE (Pinheiro et al., 2014; Suzuki et al., 2018). Insulin resistance is recognized as one of the high risks of PE since high levels of insulin in early pregnancy are directly toxic to trophoblast cells, leading to DNA damage and apoptosis, while MET (0.01 mM 24 h or 48 h) reduced primary trophoblast apoptosis and DNA damage and promoted cell survival (Vega et al., 2019). Matrix metalloproteinase (MMP) family is involved in the degradation of the extracellular matrix, which can enhance the trophoblast invasion. Among them, the role of MMP-2 and MMP-9 is particularly prominent (Belo et al., 2015). MET (20 mg/kg/d) was reported to elevate the placental MMP-2 level in high-fat diet–induced PE mice to improve shallow placental implantation and eventually ameliorate the maternal and fetal outcomes of preeclamptic mice (Wang et al., 2019). Reports concerning the positive effects of MET on the trophoblast cells include the most convincing evidence that MET can be effective in preventing and treating PE. However, there are now too few relevant reports with some contradictions between the results. Han et al. (2015) found that MET (0.5 mM) could partially reduce the inflammatory damage to trophoblast cells caused by high glucose but had no effect on improving the inhibition of angiogenesis or migration ability of trophoblast cells. Correia-Branco et al. (2018) even found that MET (1 mM, 24 h) inhibited the proliferation and migration of HTR-8/SVneo cells. Therefore, the protective effect of MET on trophoblast cells seems promising; however, it requires careful consideration, caution, and further study.

### The Promotion of the Angiogenic Status by Metformin

As mentioned in the preeclamptic pathophysiology, imbalanced secretion of angiogenic and antiangiogenic factors from the ischemic placenta results in dysplasia of the placental vessel network, systematic endothelial dysfunction, disturbance of vasomotion, and impaired ability to endothelial repair (Soobryan et al., 2018). Accordingly, PE seems to be not merely a placental disease but also a kind of cardiovascular disease in pregnancy. MET was currently the only hypoglycemic drug with evidence of cardiovascular benefits recommended by the American Association of Clinical Endocrinologists (AACE) (Garber et al., 2013), with its favorable cardioprotective effects being reported in numerous clinical trials (Luo et al., 2019). Thus, it is fascinating to discuss whether MET could have a role in the prevention or treatment of PE via its cardiovascular protection.

VEGF, a familiar angiogenic factor in endothelial cells, not only plays an essential role in placental angiogenesis and vascular recasting but also participates in infiltration, proliferation, and differentiation of trophoblast cells (Brouillet et al., 2012). VEGF is indispensable for a healthy pregnancy but declines in the maternal serum and placenta of preeclamptic women (Jena et al., 2020). MET (10 μM) was found to augment the expression of VEGF to exert proangiogenesis effects under hypoxia and hyperglycemia (Bakhashab et al., 2016). Recently, it was reported that MET (20 mg/kg) significantly reversed the decrease of the placental VEGF level on the PE-like mouse model induced by a high-fat diet and, at the same time, improved the development of placental labyrinth and fetal vascular, thus ameliorating preeclamptic symptoms in the PE-like model such as maternal blood pressure and urine protein and improving pregnancy outcomes.

sFlt-1, a representative type of antiangiogenic factor secreted primarily by syncytiotrophoblasts into maternal circulation (Karumanchi, 2016), is typically implicated in restricted vascularization, endothelial dysfunction, and elevated maternal blood pressure via decreasing the bioavailability of free VEGF and PIGF (Tomimatsu et al., 2019). Additionally, the maternal serum concentration of sFlt-1 is directly proportional to the severity of PE, and the sFlt-1/PlGF ratio is of significant clinical value for the short-term prediction in women with suspected PE (Bian et al., 2019). sENG, another typical type of placenta-derived antiangiogenic factor, inhibits the formation of capillary tubes in vitro and induces hypertension and vascular permeability in vivo, thus, in concert with sFlt-1, inducing severe vascular compromise in PE (St-Jacques et al., 1994; Li et al., 1999; Toporsian et al., 2005; Venkatesha et al., 2006). Brownfoot et al. have been researching for years whether MET could have a role in the prevention and treatment of PE (Brownfoot et al., 2016). They firstly reported that MET (0, 1, 2, and 5 mM) reduced the production of sFlt-1 and sENG dose-dependently in endothelial cells, villous cytotrophoblast cells, and preterm preeclamptic placental villous explants, the underlying mechanism of which was that MET inhibits mitochondrial electron transport chain activity upregulated in the preterm preeclamptic placenta. Also, MET (5 mM) reversed the impairment of vascular relaxation induced by incubating the maternal blood vessels obtained from the omentum at the time of cesarean sections with conditioned media of preeclamptic placentas. Furthermore, MET (1 mM) improved whole blood vessel angiogenic sprouting from omental vessel explants impaired by sFlt-1. Those results suggest that MET can reduce placental sFlt-1 and sENG secretion, ameliorate key features of endothelial dysfunction specific to PE, and enhance angiogenesis. Thus, MET is expected to be a potential preventative or therapeutic agent for PE. Consequently, with the discovery of more novel treatments, Brownfoot et al. further reported that combining MET and esomeprazole was additive in reducing expression of sFlt-1 e15a mRNA isoform and sFlt-1 secretion in primary cytotrophoblast (MET: 125 μM; esomeprazole: 25 μM), placental explants (MET: 500 μM; esomeprazole: 25 μM), and endothelial cells (MET: 1,000 μM; esomeprazole: 25 μM) (Kaitu’u-Lino et al., 2018). Nevertheless, no additive decline in sENG was observed with the combination therapy. Recently, Brownfoot et al. reported that a low-dose combination of MET and sulfasalazine reduced sFlt-1 and sENG secretion and, on the contrary, increased VEGF alpha expression in cytotrophoblast (MET: 200 μM; sulfasalazine: 200 μM) and placental explants (MET: 400 μM; sulfasalazine: 400 μM), providing a more effective treatment or prevention for PE compared to MET as a single agent (Brownfoot et al., 2020). In an obese mouse model of sFlt-1–induced PE, MET (300 mg/kg/day) significantly lowered mean and systolic blood pressure and reduced sFlt-1 levels in obese mice overexpressing sFlt-1, indicating the potential of MET in preventing PE in obese women (Jelliffe et al., 2019).

The healthy endothelium plays a vital role in maintaining vascular integrity, coagulation and fibrinolysis function, leukocyte and platelet adherence, and inflammatory responses (Brownlee, 2005). Endothelial dysfunction is regarded as one of the hallmark features of PE, resulting in generalized vasoconstriction and lessened perfusion to multiple organs. Moreover, preexisting risks such as obesity, diabetes, and poor nutrition impair endothelial function, thus exacerbating the maternal response to factors derived from the ischemic placenta (Brennan et al., 2014). For the past few years, the excellent endothelial protection of MET has been proved in diabetes mellitus, while current evidence suggests that MET also exhibits vascular endothelial protective effects in a glucose-independent manner (de Aguiar et al., 2006). Therefore, apart from the above-mentioned reduction of the sFlt-1 and sENG by MET in PE, it is plausible to say that some other mechanisms of MET having effects on protecting against endothelial dysfunction may also hold for preventing or treating PE.

NO is a well-known signaling molecule produced by the enzymatic conversion of l-arginine to l-citrulline by endothelial nitric oxide synthase (eNOS) on villous endothelial cells. NO is frequently involved in angiogenesis and spiral artery remodeling, achieving the local drop of vascular resistance to preferentially shunt blood to the uteroplacental unit (Osol and Moore, 2014). Downregulated production of NO, reduced quantification and bioavailability of eNOS, and disturbances in NO-related signaling pathways all have long been found in PE (Kim et al., 2006; Laskowska et al., 2011; Hu et al., 2019b). However, the administration of glyceryl trinitrate, an NO mimetic, was able to improve the altered uteroplacental perfusion via reducing the spiral artery resistance (Cotechini et al., 2014) in the preeclamptic rat model induced by lipopolysaccharide (LPS), contributing to the amelioration of maternal mean arterial pressure and FGR. Analogously, a drug that can increase NO production may be a potential candidate for the treatment or prevention of PE. MET has been found to have effects on the eNOS-NO system. Reportedly, therapeutically relevant concentrations of MET (50–500 μM) dose-dependently increased NO production via upregulating eNOS phosphorylation and restored impaired eNOS–HSP90 interaction in endothelial cells, which had a strong link with the AMPK activation, thus increasing NO-mediated arterial dilatation in endothelial cells in vitro (Davis et al., 2006). It was also reported that, via computational modeling and experimental validation (Cuyàs et al., 2018), MET was able to directly activate sirtuin 1 (SIRT1), which is declined in trophoblasts of patients with PE in the last trimester of pregnancy compared to normal pregnancy (Broady et al., 2017). The MET-enhanced (100 mg/kg) NO bioavailability may be mediated via the modulation of SIRT1 to increase eNOS deacetylation, resulting in the promotion of angiogenesis and the decline in endothelial cell apoptosis (Kinaan et al., 2015). Likewise, another gasotransmitter that also plays a vital role in maternal vascular adaptation to pregnancy is hydrogen sulfide (H_2_S) (Zullino et al., 2018), the production of which is catalyzed by cystathionine-beta-synthase (CBS) and cystathionine-gamma-lyase (CSE). Decreased plasma H_2_S (Wang et al., 2013) and decreased placental CBS and CSE expressions have been reported in PE (Hu et al., 2015). In the sFlt-1 rat model, reduced H_2_S productive capacity in pregnancy was inversely associated with sFlt-1 production, and exogenous H_2_S administration by NaHS (an H_2_S donor) treatment reduced symptoms of PE and returned sFlt-1 levels to normal (Holwerda et al., 2014), suggesting the great potential of H_2_S administration as a preventive and therapeutic agent for PE. In clinical practice, the closest existing compound to H_2_S is sodium thiosulfate, which has been mainly used for the treatment of calciphylaxis and resulted in case reports of severe anion gap metabolic acidosis. However, administration of MET (2.5, 5, and 10 μM) is followed by the increase of H_2_S tissue concentrations in the mouse brain, heart, kidney, and liver (Wiliński et al., 2013) and alleviates atherosclerosis via regulating CSE expression to promote H_2_S production (Ma et al., 2020), implying that MET may serve as an H_2_S donor. Currently, the NO precursor arginine (Dorniak-Wall et al., 2014; Gui et al., 2014) and the H_2_S donor (Cindrova-Davies, 2014) are both recognized as emerging therapies for PE, though their definitive benefits have not been established due to the sample size and the present study design (Burton et al., 2019). Likewise, it is well worth paying more attention to MET since it has the capacity to elevate the production of both NO and H_2_S in vivo, even though further studies are warranted to explore its potential benefit in the prevention and treatment of PE.

Endothelial progenitor cells (EPCs) are endothelial cell precursors (Burger and Touyz, 2012) that contribute to vascular remodeling and endothelial hemostasis through incorporation into vessel walls, excretion of paracrine hormones, and subsequent stimulation of angiogenesis (Asahara et al., 1997). It has been widely studied that the number and functions of EPCs are reduced in diseases associated with endothelial dysfunction such as cardiovascular diseases and diabetes mellitus (Liu et al., 2017). Recent studies demonstrated that EPCs were involved in placental development and vascular integrity during pregnancy (King et al., 2013), whereas lower quantification (Sipos et al., 2013) and reduced vasculogenic capacities (Liu et al., 2015) of EPCs were found in patients with PE (Sugawara et al., 2005) compared to the normal controls (Liu et al., 2016). Transplantation of EPCs derived from normal pregnancy ameliorated placental perfusion in preeclamptic rats (Zhu et al., 2015). Hence, a medication that has promotive effects on EPCs may have a role in preventing or treating PE. Relevant studies have focused on the effects of MET on EPCs in patients with diabetes up until now and found that patients with type 2 diabetes treated with MET had a significantly increased number of circulating EPCs (Chen et al., 2010; Liao et al., 2010). Beyond that, MET (250 mg/kg/day) improved the angiogenic abilities of EPCs, thus accelerating the wound healing process (Han et al., 2017) and stimulating angiogenesis in diabetic mice via activating the AMPK/eNOS pathway (Yu et al., 2016). MET (0.5, 1, and 2 mM) also inhibited the expression of biomarkers of fibrosis of EPCs induced by transforming growth factor β1 in vitro (Han et al., 2019). Palmitic acid (PA) is one of the most common saturated free fatty acids (FFAs) in human beings, the circulation level of which is significantly increased in preeclamptic maternal serum. PA was able to inhibit EPCs proliferation, migration, and ability of tube formation (Guo et al., 2008), while these effects of PA on EPCs were attenuated by treatment with MET (50 μM). Nevertheless, another clinical trial draws the opposite conclusion that the number and bioactivity of EPCs showed a reduced trend after treatment with MET (10 mM) (Asadian et al., 2018). The different effects of MET on EPCs among these studies may be attributed to the different concentrations of MET used. Regrettably, in reviewing the previous literature, limited data were found on the association between MET and EPCs in PE, most likely to be an emerging research direction.

### Anti-Inflammatory Effects of Metformin

PE has been described as a highly inflammatory state, with low levels of anti-inflammatory molecules and high levels of proinflammatory factors and the activation of various inflammation-related signaling pathways in both the local fetoplacental unit and maternal plasma (Tenório et al., 2019). Furthermore, aberrant maternal inflammation causes the local release of free radicals by the placenta, which results in the placenta-borne oxidative/nitrosative stress (Cotechini and Graham, 2015; Aouache et al., 2018). Recent preclinical and clinical studies have pointed out that MET not only ameliorates inflammation through the improvement of its metabolic parameters but also has a direct anti-inflammatory action. The latent mechanism of the direct anti-inflammation effects by MET may be related to the inhibition of the NF-κB pathway (Li et al., 2009). Isoda et al. (2006) found that MET inhibited the activation of NF-κB in endothelial cells and vascular smooth muscle cells concentration-dependently in vitro, thus inhibiting the secretion of inflammatory cytokines induced by interleukin-1β. Furthermore, the inhibition of the NF-κB pathway exerted by MET is closely related to the AMPK pathway (Kim et al., 2014). Hattori et al. (2006) showed that MET (2–10 mM) suppressed the TNF-α–induced endothelial cell inflammation via AMPK-dependent inhibition of the IKK/IκBα/NF-κB pathway and consequently downregulated TNF-α–induced gene expression of VCAM-1, E-selectin, ICAM-1, and MCP1, which are the typical inflammatory cytokines and adhesion molecules excessively expressed in PE. MET (100 mg/kg/d) also increased NO bioavailability by inhibiting ER stress and ROS generation via the AMPK/PPARδ pathway, contributing to the improved endothelium-dependent relaxation to show the endothelial protective benefits (Cheang et al., 2014). In the placenta of the LPS-induced PE rat model (Hu et al., 2019a), MET (300 mg/kg/d) inhibited the activation of the NF-κB pathway, reduced the release of inflammatory factors such as TNF-α and IL-6, and increased superoxide dismutase (SOD) content to reduce the generation of ONOO-. Therefore, MET inhibited oxidative stress and nitrosative stress in the placenta, reduced hypertension and proteinuria in PE rats, and improved fetal rat growth restriction and stillbirth rates. Those results suggested that MET may have a certain therapeutic effect on PE through anti-inflammatory benefits and inhibition of oxidative stress. Except for the multiple viscera or tissue damage by inflammatory cytokines, excessive inflammation is strongly linked with endothelial dysfunction. Vascular cell adhesion molecule 1 (VCAM1) is expressed by dysfunctional endothelial cells in PE, the expression of which was downregulated by MET (0, 1, 2, and 5 mM) or by combining low-dose MET (1,000 μM) and esomeprazole (25 μM) in endothelial cells stimulated by incubation with TNF-α, a cytokine upregulated in the circulation of patients with PE (Brownfoot et al., 2016; Kaitu’u-Lino et al., 2018). The important role of placental inflammation and oxidative stress in the pathophysiology of PE provides a rationale for MET as a therapy of anti-inflammatory agents and antioxidants. Recently, MET was reported to dose-dependently modify long noncoding RNA (lncRNA) H19 methylation to inhibit lncRNA H19 expression, thus modulating microRNA- (miR-) 148a-5p/P28 and miR-216-3p/EBI3 signaling pathways, respectively, ultimately leading to the reduction of IL-27, TNF-α, and IL-6 in both preeclamptic rat models and trophoblast cells (Shu et al., 2019), getting us to pay more attention to new mechanistic insights into the epigenetic effects of MET for preventing and curing PE.

## Conclusion

**FIGURE 2:**
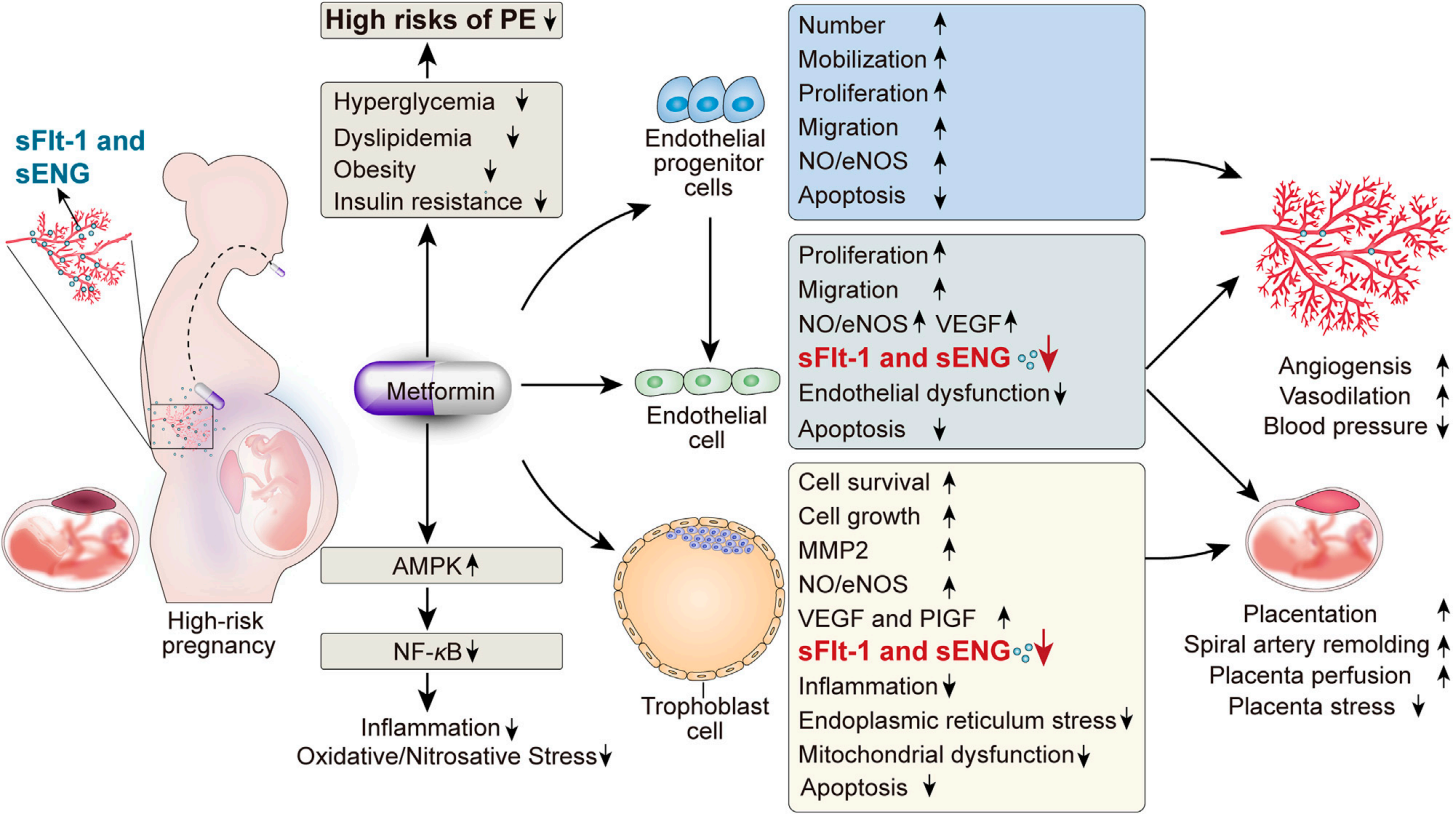
Beneficial pleiotropic effects of MET on PE and the potential mechanisms.

Even though the preeclamptic pathophysiology has been studied for almost 200 years, it remains poorly understood, thus limiting the development of preventive and therapeutic interventions of PE. Due to the complexity and diversity of the etiopathogenesis of PE, more emerging therapies should be proposed to prevent and ameliorate this health hazard. Dubbed as the “wonder drug” of the current time, MET is reported to reduce the incidence of gestational hypertension and PE according to the clinical trial results available. In terms of the underlying mechanisms, MET itself has significant effects on the improvements of hyperglycemia, dyslipidemia, obesity, and insulin resistance, which are all verified as PE-related high risks. Furthermore, there are other plausible explanations of the preventive and therapeutic effects of MET on PE, including the promotion of angiogenesis, endothelial protection, anti-inflammatory effects, and particularly the protective effects on trophoblasts against risk factors, which are beneficial to placental development (Figure 2). While the research area of the MET benefits on PE seems attractive, it is necessary to clarify that there remain huge evidence gaps between the current studies to the future clinical use of MET for the management of PE, such as the lack of relative experimental and clinical trials, the flaws existing in both the current experimental design and clinical trial protocol, the need for further studies on both short-term and long-term risks and benefits on the fetus, and the lack of high-quality evidence. Hence, determining the existing problems and exploring the corresponding solutions will be of guiding significance to the future studies of MET in PE.

Concretely, one problem is that the dosages of MET differ from each other in several reported experiments, leading to contradictory effects on the cellular bioactivities, such as the protective benefits on trophoblast with low-dose MET and the impairment with high dose. Thus the dose of MET in different experiments is supposed to be limited within a certain range. To tailor the dose of MET in future experiments, a proper understanding of the pharmacokinetics of MET during pregnancy, especially the placental MET concentration, is essential. In nonpregnant patients, the plasma concentration range of MET after liver metabolism is usually 10–40 μM with the oral administration of 0.5–2.5 g/d (He and Wondisford, 2015). However, due to the specificity of drug absorption, metabolism, and transport during pregnancy, the gestational plasma concentration range of MET should be reconsidered, although the exact range is yet unclear. Considering the limited researches available, we find that pregnancy significantly increases the bioactivity of MET, which may partly be due to the elevated levels of progesterone during pregnancy prolonging small intestine transit time to increase the absorption of MET. Besides, the renal clearance and secretion clearance of MET are both higher in pregnancy because of the enhanced renal plasma flow in pregnant women (Hughes et al., 2006). Pregnancy also increases the apparent oral clearance (CL/F) of MET, indicating that higher initial doses may be needed during gestations. Intriguingly, increasing the dose of MET during pregnancy resulted in lower bioactivity but higher CL/F, suggesting that choosing an appropriate dose of MET during pregnancy remains a clinical dilemma that needs to be further studied (Liao et al., 2020). MET can freely cross the placenta with concentrations that can be half or more than the maternal plasma concentration (Vanky et al., 2005). As for infant exposure to MET, the subtherapeutic doses of MET in breast milk are not expected to cause pharmacological effects (Eyal et al., 2010). Nevertheless, almost all previous studies were conducted with suprapharmacological concentrations (doses) of MET, much higher than maximally achievable therapeutic concentrations, and the results of those experiments may be too controversial to truly reflect the effects of MET in the human body. Therefore, further exploring the range of both plasma and placental concentration after metabolic transport of maternal MET during pregnancy should be the top priority in the future, which can guide the follow-up experiments to set the drug concentration reasonably. Subsequently, through serial concentration gradient, it is expected that the best dosage of MET is selected that exhibits the maternal and fetal protective benefits with minimal adverse effects, finally guiding the clinical medication.

Another unsolved problem is whether the MET is effective for every subtype of PE. At present, scholars have reached a consensus that PE is more of a syndrome with various subtypes rather than a single disease. Consequently, the possible prevention and treatment exerted by MET on PE need to be further verified in a variety of preeclamptic animal models; this could be helpful in further defining the subclassifications and subtypes of PE for which MET may be applicable (Cushen and Goulopoulou, 2017). Similarly, relevant prospective population studies and RCTs need to be conducted, which will also allow us to identify a subset of patients who may benefit from the administration of MET.

Last but not least, there is an urgent need for more clinical trials related to the preventive and therapeutic effects of MET on PE, particularly the clinical trials designed to evaluate the reduction in the prevalence of PE as a primary endpoint. Currently, PE is a secondary outcome in most existing studies, suggesting that MET is temporarily not recommended for the clinical prevention of PE, referring to the 2019 ACOG notice (ACOG, 2019a). Besides, relevant clinical trials have focused on the preventive effects of MET on HDP for years; however, its therapeutic effects have so far proved inconclusive. Reportedly, Cluver et al. are working on a randomized, double-blind, placebo-controlled, phase II trial (PI2 trial) to evaluate the effect of MET on treating preterm PE (Cluver et al., 2019). In the PI2 trial, the researchers intend to recruit 150 women with preterm PE at the gestational age of 26^+0^ to 31^+6^ weeks. Participants will randomly receive either 3 g of MET or placebo daily; the time from randomization until delivery and the maternal and neonatal outcomes will be exploratory. The PI2 clinical trial can fill the current research gap and have a guiding significance in the design of more and larger prospective clinical studies to verify the efficacy of MET in the treatment of PE in the future. The changes of key molecular indicators in serum, placenta, umbilical cord, and other clinical specimens should be simultaneously detected. Moreover, clarifying the relationship between MET and HDP with the combination of the basic experiments and clinical data will be very helpful.

In summary, MET may be a reasonable alternative approach to prevent and treat HDP, but it cannot be ignored that there are still unsettled questions. We are looking forward to more related studies to be reported in the future, which can bring MET from experimental research to clinical use.

## Author Contributions

YZ and XL drafted the manuscript by reviewing the literature. LY participated in the discussion and prepared the chart. The corresponding author LZ guided the formation of the entire manuscript. All authors contributed to the article and approved the submitted version.

## Funding

This work was supported by the National Nature Science Foundation of China (No. 81741002 to Li Zou and No. 81703242 to Qingqing Luo).

## Conflict of Interest

The authors declare that the research was conducted in the absence of any commercial or financial relationships that could be construed as a potential conflict of interest.
